# Risk of immune-mediated inflammatory diseases in newly diagnosed ankylosing spondylitis patients: a population-based matched cohort study

**DOI:** 10.1186/s13075-019-1980-1

**Published:** 2019-08-29

**Authors:** Hsin-Hua Chen, Wen-Cheng Chao, Yi-Hsing Chen, Tsu-Yi Hsieh, Kuo-Lung Lai, Yi-Ming Chen, Wei-Ting Hung, Ching-Tsai Lin, Chih-Wei Tseng, Ching-Heng Lin

**Affiliations:** 1 Department of Medical Research, Taichung Veterans General Hospital, Taichung, Taiwan; 2 Division of Allergy, Immunology, and Rheumatology, Department of Internal Medicine, Taichung Veterans General Hospital, Taichung, Taiwan; 3 School of Medicine, National Yang-Ming University, Taipei, Taiwan; 4 Institute of Biomedical Science and Rong-Hsing Research Center for Translational Medicine, Chung-Hsing University, Taichung, Taiwan; 5 Institute of Public Health and Community Medicine Research Center, National Yang-Ming University, Taipei, Taiwan; 6 Department of Industrial Engineering and Enterprise Information, Tunghai University, Taichung, Taiwan; 7 Division of Chest Medicine, Department of Internal Medicine, Taichung Veterans General Hospital, Taichung, Taiwan; 8 Department of Nursing, College of Medicine & Nursing, Hung Kuang University, Taichung, Taiwan; 9 Department of Medical Education, Taichung Veterans General Hospital, Taichung, Taiwan; 10 Ph.D. Program of Business, Feng Chia University, Taichung, Taiwan; 11 Graduate Institute of Biomedical Electronics and Bioinformatics, National Taiwan University, Taipei, Taiwan; 12 Department of Healthcare Management, National Taipei University of Nursing and Health Sciences, Taipei, Taiwan

**Keywords:** Ankylosing spondylitis, Immune-mediated inflammatory disease, Incidence

## Abstract

**Objective:**

To investigate the risk of immune-mediated inflammatory diseases (IMIDs) in patients with ankylosing spondylitis (AS).

**Methods:**

Using 2003–2012 claims data from the Taiwanese National Health Insurance Research Database, we identified 30,911 newly diagnosed AS patients requiring medical therapy from 2006 to 2012. In addition, we randomly selected 309,110 non-AS individuals matching (1:10) the AS patients with regard to age, sex and the year of the index date. After excluding subjects with the corresponding prior IMIDs, we calculated the incidence rates (IRs) of various IMIDs in the AS and non-AS cohorts and estimated the hazard ratios (HRs) with 95% confidence intervals after adjusting for age, sex, the Charlson comorbidity index, the frequency of ambulatory visits during the follow-up period and medications. We conducted sensitivity analyses by excluding those who developed IMIDs within 3 months after the index date.

**Results:**

In the follow-up period, we found that newly diagnosed AS patients had significantly increased risks of acute anterior uveitis, psoriasis, Sjögren’s syndrome, thromboangiitis obliterans, Behcet’s disease and sarcoidosis. However, the risk of Sjögren’s syndrome did not increase in AS patients in the sensitivity analysis. In the same period, this study found no significant differences in the risks of Crohn’s disease, ulcerative colitis, systemic lupus erythematosus, systemic sclerosis, dermatomyositis, polymyositis, pemphigus and vitiligo between newly diagnosed AS patients and non-AS individuals. AS patients had a significantly reduced risk of rheumatoid arthritis.

**Conclusion:**

Newly diagnosed Taiwanese AS patients had increased risks of acute anterior uveitis, psoriasis, thromboangiitis obliterans, Behcet’s disease and sarcoidosis, but a reduced risk of rheumatoid arthritis.

**Electronic supplementary material:**

The online version of this article (10.1186/s13075-019-1980-1) contains supplementary material, which is available to authorized users.

## Background

Immune-mediated inflammatory disease (IMID) is a term that covers a group of disorders characterised by altered immune regulation causing chronic inflammation in targeted organs or systems. IMIDs affect around 3 to 7% of the population with an estimated incidence of 80 per 10^5^ person-years [1–3]. Accumulating data from genome-wide association studies have revealed a genetic overlap among IMIDs [4]. Previous studies have shown that a patient with IMID has an increased risk of developing another IMID [5–7]. Ankylosing spondylitis (AS) is an IMID that mainly affects the axial skeleton and sometimes affects the peripheral joints and anthesis region [8], with a prevalence of 0.11 to 0.38% in Taiwan [9, 10]. In recent years, AS has been considered as a prototype of spondyloarthritis (SpA) that has been found to be associated with other IMIDs with shared immunopathogenesis referred to as extra-articular manifestations. Among the SpA concept-related extra-articular manifestations, acute anterior uveitis (AAU), psoriasis and inflammatory bowel disease (IBD), including Crohn’s disease (CD) and ulcerative colitis (UC), are most strongly associated with AS [11]. Several previous epidemiological studies reported the incidence and prevalence of extra-articular manifestations among AS patients [12, 13]. However, to our knowledge, no prior study had concurrently assessed the risks of developing various IMIDs in addition to SpA concept-related IMIDs (i.e. AAU, psoriasis, CD and UC) among AS patients.

The Taiwanese National Health Insurance Research Database (NHIRD) has been used to conduct population-based epidemiologic studies. The present study aimed to investigate the incidences of various IMIDs, including SpA concept-related EAMs (i.e. AAU, psoriasis, CD and UC), systemic lupus erythematosus (SLE), Sjögren’s syndrome (SS), rheumatoid arthritis (RA), systemic sclerosis (SSc), polymyositis (PM), dermatomyositis (DMtis), thromboangiitis obliterans (TAO), Behcet’s disease (BD), sarcoidosis, pemphigus and vitiligo in a Taiwanese nationwide AS cohort compared with a matched non-AS cohort, using the NHIRD.

## Methods

### Study design

This was a retrospective matched cohort study.

### Data source

The study data were the 2003–2012 claims data from the NHIRD. In 1995, Taiwan initiated a compulsory National Health Insurance program that currently covers over 99% of the Taiwanese population. The data of the NHIRD includes comprehensive claims data regarding medication prescription, ambulatory care services, admission services and traditional medical services. Some personal data and history data, such as body weight, body length, alcohol use and smoking, are not available in the NHIRD. The Bureau of National Health Insurance has improved the accuracy of the claims data in the NHIRD by checking original medical records regularly [14]. The National Health Research Institute manages the NHIRD and provides the database to researchers for research purpose after anonymisation of personal information.

We utilised multiple NHIRD datasets in this study, including 2003–2012 outpatient and inpatient claims data and enrollment data. We selected all newly diagnosed AS patients from 2006 to 2012 as the study cohort. The National Health Research Institute randomly selected one million individuals who were enrolled in 2000 to build a representative longitudinal health insurance database. We selected the comparison cohort from the representative population in this longitudinal database, from which we extracted 2003–2012 claims data for analysis.

For patients with severe or major diseases, such as malignancy, SLE, SSc, RA, PM, DMtis, TAO, BD, pemphigus, SS, CD and UC, the Bureau of National Health Insurance established a registry of catastrophic illness patients. Those who had a catastrophic illness certificate were exempt from copayment for all services with a corresponding catastrophic illness diagnosis. However, a catastrophic illness certificate was only issued after validation by at least two qualified specialists through a comprehensive review of the original medical charts.

### Definition of AS

AS patients were considered as those having at least three ambulatory visits with a diagnosis of AS (International Classification of Diseases, Ninth Revision, Clinical Modification [ICD-9-CM] code 720.0) and a concurrent prescription of nonsteroidal anti-inflammatory drugs (NSAIDs), sulfasalazine (SSZ), methotrexate (MTX) or corticosteroids from 2003 to 2012. In Taiwan, AS was diagnosed according to the modified New York criteria for AS proposed in 1984 [15].

### Study subjects

**Fig. 1:**
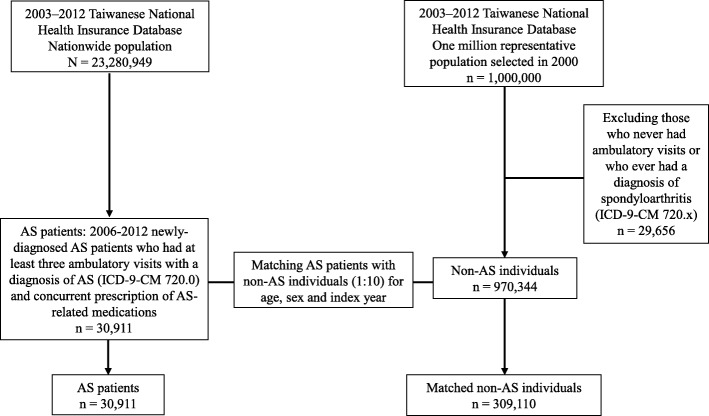
Flow chart of study subject enrollment

The flow chart of study subject enrollment is shown in Fig. 1.

### AS patients newly diagnosed in 2006–2012 from the entire Taiwanese population

This study included all newly diagnosed AS patients from 2006 to 2012. We excluded patients who had one or more inpatient or outpatient visits with a diagnosis of AS before 2006. We defined the index date for AS patients as the first date of outpatient or inpatient visit with a diagnosis of AS.

### Matched non-AS comparison group selected from the one million nationally representative individuals in Taiwan

Non-AS individuals were considered as those having no ambulatory or inpatient diagnosis of ICD-9-CM 720.x from 2003 to 2012. We randomly selected non-AS individuals matching (1:10) the AS patients for age, sex and the year of the index date (index year) from the one million representative population in Taiwan. The time of the first ambulatory visit in the index year for any reason was used as the index date for the non-AS group.

### Outcome

The outcome was the time from the index date to the time of the first outpatient or inpatient visit with the diagnosis of various IMIDs. If the IMIDs were included in the catastrophic illnesses, patients who developed these IMIDs were considered to be those who had a catastrophic illness certificate for the corresponding ICD-9-CM diagnosis (SLE, 710.0; SS, 710.2; RA, 714.0; SSc, 710.1; PM, 710.4; DMtis, 710.3; TAO, 443.1; BD, 136.1; CD, 555; UC, 556; pemphigus, 694.4) after the index date. Patients were considered to have sarcoidosis if they had at least three outpatient visits or one inpatient visit with the corresponding ICD-9-CM diagnosis (sarcoidosis, 135). Patients were considered to have AAU if they had at least three outpatient visits or one inpatient visit involving ophthalmologists with the ICD-9-CM diagnosis code 364.00–364.02, 364.04–364.05 or 364.3. Patients were considered to have psoriasis or vitiligo if they had at least three outpatient visits or one inpatient visit involving dermatologists with the corresponding ICD-9-CM diagnosis (psoriasis, 696.1; vitiligo, 709.01). We considered the censored date as 31 December 2012 (the last date of the data used in this study) or the time of withdrawal from the National Health Insurance for various reasons, such as leaving and death, whichever came first. The follow-up duration was from the index date to the date of IMID occurrence or from the index date to the censor date. We calculated the incidences of various IMIDs by dividing the number of incident IMID cases by the sum of the follow-up durations among those who had no outpatient or inpatient visit with the corresponding IMID diagnosis before the index date.

### Sensitivity analysis

To reduce the possibility that various IMIDs occurred concurrently with or earlier than AS, only patients who developed IMIDs more than 3 months after the index date were considered the incident IMID patients. We selected 3 months as the cut-off time for sensitivity analysis, given that patients usually could only arrange an outpatient appointment within 3 months in Taiwan. Therefore, if a patient suffered from various IMID-related symptoms, the diagnoses of the IMIDs may be delayed for up to 3 months.

### Potential confounders

The associations between AS and the risks of various IMIDs were adjusted for potential confounders, including sex, age at the index date, comorbidity within 1 year before the index date, the frequency of ambulatory visits for any reason and concurrent medication use. We considered the matching variables (i.e. age and sex) as covariates in the multivariable Cox regression analyses because we also adjusted for additional confounders in the study [16]. We considered concomitant medications as potential confounders because they may influence the risk of IMID through immunomodulatory effects. Concurrent medications included NSAIDs, corticosteroids, MTX, SSZ, leflunomide, hydroxychloroquine and immunosuppressants (i.e. cyclosporine, azathioprine, cyclophosphamide, mycophenolate or mycophenolic acid). Because multisystem involvement of IMIDs is not uncommon, patients who had comorbidies might have an increased chance to receive investigations for IMIDs, leading to detection bias. Therefore, we also considered comorbidities as potential confounders. We used the Charlson comorbidity index (CCI) (i.e. 0, ≥ 1) adapted by Deyo et al. [17] to indicate the general comorbid conditions. The presence of diseases for calculating the CCI was considered as having at least three outpatient visits or at least one hospitalisation with the corresponding ICD-9-CM diagnosis within 1 year before the index date. We also considered the frequency of ambulatory visits for any reason as potential confounder given that a higher frequent visit number may introduce a greater chance to survey another disease, leading to detection bias.

### Statistical analysis

Continuous variables are presented as a mean ± standard deviation, and categorical variables are presented as the percentage of patients. We examined differences in continuous variables using Student’s *t* test and differences in categorical variables using Pearson’s *χ*^2^ test. We quantified the associations between AS and the risks of various IMIDs by estimating hazard ratios (HRs) with 95% confidence intervals (CIs) using multivariable Cox proportional regression analysis after adjusting for potential confounders. We used six models based on the number of covariates (i.e. no adjustment; age; age and sex; age, sex and medications; age, sex, medications and the frequency of ambulatory visits; age, sex, medications, the frequency of ambulatory visits and CCI) to estimate HRs with 95% CI for the risks of IMIDs. We calculated the Akaike information criterion (AIC) for each model and selected the final models with the best model fit according to AIC (i.e. the least AIC) [18]. If two or more models had the same AIC, we selected the model with the greatest number of covariates. All statistical analyses by SAS statistical software, version 9.3 (SAS Institute, Inc., Cary, NC, USA). We considered a two-tailed *P* value < 0.05 as statistically significant.

## Results

### Baseline characteristics

The study included 30,911 newly diagnosed AS patients in the AS group and 309,110 non-AS individuals matching (1:10) the AS patients with regard to age, sex and the year of the first AS diagnosis date (index date) in the comparison group. The baseline characteristics of both groups are presented in Table 1. In both groups, the mean (± SD) age was 42 ± 17 years and 62.9% of the patients were male. The proportion of patients with a CCI of ≥ 1 was higher in the AS group than in the non-AS group. Additionally, the proportions of patients with histories of uveitis, psoriasis, UC, SLE, SS, RA, SSc, DMtis, TAO, BD, sarcoidosis and vitiligo were higher in the AS group than in the non-AS group. However, the proportions of patients with histories of CD, PM and pemphigus were not different between the AS and non-AS groups.

**Table 1:** Demographic data and clinical characteristics of the study subjects

	Non-AS	AS	
	(*n* = 309,110)	(*n* = 30,911)	*P* value
Age, years (mean ± SD)	42 ± 17	42 ± 17	1.00
Sex			1.00
Female	114,560 (37.1)	11,456 (37.1)	
Male	194,550 (62.9)	19,455 (62.9)	
CCI (mean ± SD)	0.22 ± 0.74	0.35 ± 0.89	< 0.01
CCI group			< 0.01
0	269,864 (87.3)	24,208 (78.3)	
≥ 1	39,246 (12.7)	6703 (21.7)	
Prior IMID			
Acute anterior uveitis	604 (0.194)	1695 (5.483)	< 0.01
Psoriasis	1019 (0.330)	455 (1.472)	< 0.01
Inflammatory bowel disease	71 (0.023)	18 (0.058)	< 0.01
Crohn’s disease	41 (0.013)	7 (0.023)	0.19
Ulcerative colitis	38 (0.012)	13 (0.042)	< 0.01
Systemic lupus erythematosus	193 (0.062)	79 (0.256)	< 0.01
Sjögren’s syndrome	137 (0.044)	99 (0.320)	< 0.01
Rheumatoid arthritis	401 (0.13)	345 (1.116)	< 0.01
Systemic sclerosis	20 (0.006)	10 (0.032)	< 0.01
Dermatomyositis	12 (0.004)	7 (0.023)	< 0.01
Polymyositis	11 (0.004)	1 (0.003)	0.93
Thromboangiitis obliterans	1 (0.0003)	1 (0.003)	< 0.01
Behcet’s disease	18 (0.006)	24 (0.078)	< 0.01
Pemphigus	16 (0.005)	2 (0.006)	0.77
Sarcoidosis	13 (0.004)	6 (0.019)	< 0.01
Vitiligo	144 (0.047)	30 (0.097)	< 0.01

Data are presented as number (%) unless specified otherwise

*Abbreviations*: *AS* ankylosing spondylitis, *SD* standard deviation, *CCI* Charlson comorbidity index, *IMID* immune-mediated inflammatory disease

### Incidence rates and incidence rate ratios of various IMIDs

Table 2 shows the incidence rates of IMIDs in both groups and the incidence rate ratios (IRRs) with 95% CIs in the AS group compared with the non-AS group. Among AS patients, the three highest incidence rates were identified for AAU, RA and psoriasis. On comparing AS patients and non-AS individuals, we found that AS patients had significantly higher incidence rates of BD, RA, AAU, TAO, SS, PM, SSc, UC, sarcoidosis, CD, SLE, DMtis, psoriasis and vitiligo, but not pemphigus.

**Table 2:** Comparison of the incidence rates of developing various immune-mediated inflammatory diseases between AS patients and non-AS individuals

Autoimmune disease	Total	Event (%)	Total person-years	Incidence rate (/10^5^ years)	IRR (95% CI)
Acute anterior uveitis					
Non-AS	307,265	420 (0.14)	1,233,168	34.1	Reference
AS	28,335	643 (2.13)	103,487	583.6	17.14 (15.13–19.41)
Psoriasis					
Non-AS	307,048	508 (0.17)	1,231,732	41.2	Reference
AS	30,240	176 (0.58)	111,703	157.6	3.82 (3.22–4.53)
Inflammatory bowel disease					
Non-AS	299,243	12 (0.004)	1,205,603	1.0	Reference
AS	29,462	9 (0.03)	109,791	8.2	8.24 (3.47–19.55)
Crohn’s disease					
Non-AS	299,776	4 (0.001)	1,207,612	0.3	Reference
AS	29,560	3 (0.01)	110,132	2.7	8.22 (1.84–36.75)
Ulcerative colitis					
Non-AS	308,500	8 (0.003)	1,238,652	0.6	Reference
AS	30,793	7 (0.02)	114,074	6.1	9.50 (3.45–26.20)
Systemic lupus erythematosus					
Non-AS	308,500	40 (0.01)	1,238,580	3.2	Reference
AS	30,517	29 (0.10)	113,090	25.6	88.20 (11.03–705.18)
Sjögren’s syndrome					
Non-AS	306,049	80 (0.03)	1,230,358	6.5	Reference
AS	29,772	91 (0.31)	110,650	82.2	12.65 (9.37–17.08)
Rheumatoid arthritis					
Non-AS	305,326	112 (0.04)	1,226,848	9.1	Reference
AS	27,531	190 (0.69)	102,233	185.9	20.36 (16.12–25.71)
Systemic sclerosis					
Non-AS	309,048	9 (0.003)	1,240,674	0.7	Reference
AS	30,872	8 (0.03)	114,346	7.0	9.64 (3.72–25.00)
Dermatomyositis					
Non-AS	308,846	8 (0.003)	1,239,911	0.6	Reference
AS	30,843	4 (0.01)	114,233	3.5	5.43 (1.63–18.02)
Polymyositis					
Non-AS	308,832	2 (0.001)	1,239,818	0.2	Reference
AS	30,827	2 (0.01)	114,166	1.8	10.86 (1.53–77.10)
Thromboangiitis obliterans					
Non-AS	309,093	2 (0.001)	1,240,854	0.2	Reference
AS	30,907	3 (0.01)	114,474	2.6	16.26 (2.72–97.31)
Behcet’s disease					
Non-AS	309,039	4 (0.001)	1,240,654	0.3	Reference
AS	30,820	21 (0.07)	114,144	18.4	57.06 (19.59–166.24)
Pemphigus					
Non-AS	309,061	4 (0.001)	1,240,728	0.3	Reference
AS	30,906	2 (0.01)	114,477	1.7	5.42 (0.99–29.59)
Sarcoidosis					
Non-AS	309,088	21 (0.01)	1,240,775	1.7	Reference
AS	30,897	16 (0.05)	114,402	14.0	8.26 (4.31–15.84)
Vitiligo					
Non-AS	308,750	98 (0.03)	1,239,523	7.9	Reference
AS	30,838	21 (0.07)	114,251	18.4	2.32 (1.45–3.72)

*Abbreviations*: *AS* ankylosing spondylitis, *IRR* incidence rate ratio, *CI* confidence interval

### Multivariable Cox regression analyses for the associations between AS and the development of IMIDs after the index date

Crude and multivariable Cox regression analyses for the relative risks of various IMIDs occurring after the index date in AS patients when compared with non-AS individuals are shown in Additional file 1: Table S1. We reveal the HRs with 95% CIs in the models with the least AIC in Table 3. The risks of developing AAU, psoriasis, CD, SS, TAO, BD and sarcoidosis were increased in AS patients. On the other hand, the risk of developing RA was lower in AS patients than in non-AS individuals. However, the risks of developing SLE, PM, DMtis, SSc, pemphigus, UC and vitiligo did not differ significantly between AS patients and non-AS individuals.

**Table 3:** Multivariable Cox regression analyses for the relative risks of developing various IMIDs in AS patients when compared with non-AS individuals

IMID	IMID occurred after the index date		Excluding those with IMID within 3 months after the index date	
	HR (95% CI)	*P* value	HR (95% CI)	*P* value
Acute anterior uveitis	9.07 (7.52–10.94)^e^	< 0.01	10.98 (9.10–13.25)^e^	< 0.01
Psoriasis	1.69 (1.24–2.29)^f^	< 0.01	1.41 (1.01–1.96)^e^	0.04
Inflammatory bowel disease	2.11 (0.39–11.48)^d^	0.39	0.35 (0.06–2.06)^e^	0.24
Crohn’s disease	8.01 (1.79–35.79)^c^	0.01	2.74 (0.31–24.52)^b^	0.37
Ulcerative colitis	0.33 (0.05–2.37)^f^	0.27	0.33 (0.05–2.37)^f^	0.27
Systemic lupus erythematosus	0.93 (0.49–1.79)^f^	0.84	0.81 (0.40–1.64)^f^	0.56
Sjögren’s syndrome	2.05 (1.39–3.01)^f^	< 0.01	1.29 (0.84–1.99)^f^	0.25
Rheumatoid arthritis	0.70 (0.50–0.99)^f^	0.04	0.46 (0.33–0.65)^e^	< 0.01
Systemic sclerosis	1.65 (0.45–6.02)^e^	0.45	1.65 (0.45–6.02)^e^	0.45
Dermatomyositis	0.95 (0.22–4.02)^f^	0.94	0.29 (0.04–1.99)^f^	0.21
Polymyositis	7.96 (0.97–65.56)^f^	0.05	7.96 (0.97–65.56)^f^	0.05
Thromboangiitis obliterans	16.62 (2.78–99.52)^a^	< 0.01	16.62 (2.78–99.52)^a^	< 0.01
Behcet’s disease	20.37 (5.54–74.85)^f^	< 0.01	26.20 (6.27–109.45)^e^	< 0.01
Pemphigus	0.97 (0.07–13.48)^d^	0.98	0.97 (0.07–13.48)^d^	0.98
Sarcoidosis	5.39 (2.15–13.48)^f^	< 0.01	6.09 (2.38–15.58)^f^	< 0.01
Vitiligo	1.55 (0.73–3.32)^f^	0.26	1.42 (0.63–3.22)^f^	0.40

*Abbreviations*: *AS* ankylosing spondylitis, *IMID* immune-mediated inflammatory disease, *HR* hazard ratio, *CI* confidence interval

Adjusted variables: ^a^none; ^b^age; ^c^age and sex; ^d^age, sex and medications; ^e^age, sex, medications and the frequency of visits; ^f^age, sex, medications, the frequency of visits and Charlson comorbidity index. Models with the least Akaike information criterion were selected

### Sensitivity analysis for the risks of IMIDs excluding those with IMID within 3 months after the index date in AS patients

Crude and multivariable Cox regression analyses for the relative risks of various IMIDs excluding those with IMID within 3 months after the index date in AS patients when compared with non-AS individuals are shown in Additional file 2: Table S2. We reveal the HRs with 95% CIs in the models with the least AIC in Table 3. The risks of AAU, psoriasis, TAO, BD and sarcoidosis remained significantly higher in AS patients than in non-AS individuals. On the other hand, the risk of RA was significantly lower in AS patients compared with controls. However, the risks of SLE, PM, DMtis, SSc, pemphigus, SS, CD, UC and vitiligo were not significantly different between AS patients and non-AS individuals.

## Discussion

To our knowledge, our study is the first to concurrently estimate the relative risks of developing various IMIDs in addition to SpA concept-related IMIDs (i.e. AAU, psoriasis, CD and UC) in AS patients when compared with non-AS individuals. Considering the data of model fitting and sensitivity analyses, we found that newly diagnosed AS patients had increased risks of AAU, psoriasis, TAO, BD and sarcoidosis, but a decreased risk of RA. Consistent with the findings in previous studies [12, 19], the risks of developing SpA concept-related comorbidities, including uveitis and psoriasis, were higher in newly diagnosed AS patients compared with non-AS individuals. However, inconsistent with the findings in previous studies [12, 19, 20], we failed to demonstrate statistically significant differences in the risks of UC and CD between newly diagnosed AS patients and non-AS individuals after adjusting potential confounders. Because IBD was increased at baseline, it is likely that the failure to identify a clinical association of AS with IBD was because the onset of IBD in most cases was prior to the baseline for study entry. Other possible explanations of this inconsistency included the adjustment of many covariates, possible AS diagnosis delay and the low incidences of CD and UC in Taiwan [21].

After adjusting for potential confounders, we found a positive correlation between AS and the development of SS. However, the risk of developing SS did not significantly increase in newly diagnosed AS patients after excluding patients who developed SS within 3 months after the index date. The inconsistent finding in the sensitivity analysis might be explained by the presence of radiographic sacroiliitis in a significant proportion (24.7%) of primary SS patients reported by Eren et al. [22] and the insidious onset of SS [23].

We also found that newly diagnosed AS patients had an increased risk of developing TAO and BD. In 1999, Puéchal et al. found that 11 (12.5%) of 83 patients with TAO had initial rheumatic manifestations. Of them, two patients had HLA-B27-positive SpA and one presented with oligoarthritis [24]. In 2015, Lpoalco et al. also reported the coexistence of axial SpA and TAO in a young woman [25]. Possible explanations for the linkage of AS with TAO include shared genetic risk factors, e.g. HLA-B40 [26, 27], and environmental risk factors, e.g. smoking [28, 29]. In 1974, Moll et al. revealed associations among AS, psoriatic arthritis, Reiter’s syndrome, intestinal arthropathies and BD and suggested that SpA complex could include BD [30]. Many cases with the coexistence of AS and BD have also been reported [31–42]. A prior study investigating the skeletal manifestations in 79 BD patients found that 5% of them had definite AS [43]. There are some possible explanations for the markedly increased risk of BD in newly diagnosed AS patients. First, AS and BD had shared genetic associations with MHC class I, interleukin 23 receptor (IL23R) and endoplasmic reticulum aminopeptidase 1 (ERAP1) [44]. Second, similar inflammatory cytokine profiles in synovial fluid existed in AS and BD patients [45], indicating the similarities in the pathogeneses of AS and BD. Third, a previous study showed that articular symptoms, including spinal pain, were the first disease manifestation in 16.5% of 79 BD patients [43]. Therefore, some BD patients might be misdiagnosed as AS initially.

The present study showed that newly diagnosed AS patients had an increased risk of sarcoidosis. Wu et al. found that sarcoidosis patients had an increased risk of comorbid AS (adjusted odds ratio, 3.80; 95% CI, 2.42–5.97) [46]. We also found that the proportion of patients with a history of sarcoidosis was higher among AS patients than among non-AS individuals. Kim et al. found that sarcoidosis was associated with the *IL23R* gene [47], and it is known that AS is also associated with this gene. Of note, several case reports indicated an association between tumour necrosis inhibitors, which are used for AS therapy, and the development of sarcoidosis [48, 49]. We found a negative association between AS and the risk of RA development. This negative association may be explained by the distinct major genetic risk loci between AS (MCH class I/IL23R/ERAP1) and RA (MHC class II/PTPN22/STAT4/IRF5) [44].

The strengths of this study are the minimisation of selection bias and the inclusion of adequate incident cases with IMIDs using a nationwide population-based database. However, this study has some limitations. First, the accuracy of the diagnosis of AS according to claims data is concerning. Therefore, false positive and false negative diagnoses may occur both in the AS group and the comparison group. However, the accuracy of the diagnosis has improved following a routine inspection of the original medical records [14]. Second, the ICD-9-CM code for pemphigus (i.e. 694.4) includes different entities like pemphigus vulgaris, pemphigus foliaceous and others. Although these disease entities shared the feature of immune-mediated acantholysis, such diagnostic pooling was still of concern. Third, although we used several methods to improve the accuracy of IMID diagnoses, the detection rates of IMIDs in AS patients may not be equal to those in non-AS individuals. However, we minimised such differential detection bias by adjusting the frequency of ambulatory visits to decrease the possibility of an overestimation of the risks of IMIDs in AS patients. Fourth, some potential confounding factors, including socioeconomic status, smoking history, drinking history and family history, were not recorded in the NHIRD. Fifth, by focusing on incidence, the study design loses power to identify clinical association between AS and IBD. Finally, it might not be possible to extrapolate the study results, especially the IBD results, to non-Taiwanese populations.

## Conclusions

This nationwide, population-based, cohort study showed newly diagnosed AS patients had increased risks of AAU, psoriasis, TAO, BD and sarcoidosis and a decreased risk of RA. These significant associations indicate potential shared or distinct genetic, pathogenic and clinical relationships between AS and these IMIDs and also suggest that physicians should survey symptoms and signs related to the positively correlated IMIDs when managing AS patients. Further clinical, genetic and pathogenic studies are also necessary to elucidate the role of AS in the development of uveitis, psoriasis, TAO, BD and sarcoidosis and to identify the risk factors or predictors.

## Additional files

Additional file 1: **Table S1.** Crude and multivariable Cox regression analyses for the relative risks of various IMIDs occurring after the index date in AS patients when compared with non-AS individuals. (DOCX 23 kb)

Additional file 2: **Table S2.** Crude and multivariable Cox regression analyses for the relative risks of developing various IMIDs excluding those with IMID within three months after the index date in AS patients when compared with non-AS individuals. (DOCX 24 kb)

## Data Availability

The datasets used and analysed during the current study are available from the corresponding author on reasonable request.

## Abbreviations

AS: Ankylosing spondylitis
BD: Behcet’s disease
CCI: Charlson comorbidity index
CD: Crohn’s disease
CI: Confidence interval
DMtis: Dermatomyositis
ERAP1: Endoplasmic reticulum aminopeptidase 1
HR: Hazard ratio
IBD: Inflammatory bowel disease
ICD-9-CM: International Classification of Diseases, Ninth Revision, Clinical Modification
IL23R: Interleukin 23 receptor
IMID: Immune-mediated inflammatory disease
IRR: Incidence rate ratio
MTX: Methotrexate
NHIRD: National Health Insurance Research Database
NSAID: Nonsteroidal anti-inflammatory drugs
RA: Rheumatoid arthritis
SpA: Spondyloarthritis
SLE: Systemic lupus erythematosus
SS: Sjögren’s syndrome
SSc: Systemic sclerosis
SD: Standard deviation
SSZ: Sulfasalazine
TAO: Thromboangiitis obliterans
UC: Ulcerative colitis
