# Supplementary material for: Risk of immune-mediated inflammatory diseases in newly diagnosed ankylosing spondylitis patients: a population-based matched cohort study
Source: Arthritis Res Ther. 2019 Aug 29;21:196. doi: 10.1186/s13075-019-1980-1 (PMC6716905; doi:10.1186/s13075-019-1980-1)
Supplement: Supplementary file 1 — Table S1. Crude and multivariable Cox regression analyses for the relative risks of various IMIDs occurring after the index date in AS patients when compared with non-AS individuals. (DOCX 23 kb) [file 13075_2019_1980_MOESM1_ESM.docx]

**Risk of Immune-mediated Inflammatory Diseases in Newly-diagnosed Ankylosing Spondylitis Patients: A Population-based Matched Cohort Study**

Hsin-Hua Chen, Wen-Cheng Chao, Yi-Hsing Chen, Tsu-Yi Hsieh, Kuo-Lung Lai, Yi-Ming Chen, Wei-Ting Hung, Ching-Tsai Lin, Chih-Wei Tseng, Ching-Heng Lin

**Additional file 1.** Crude and multivariable Cox regression analyses for the relative risks of various IMIDs occurring after the index date in AS patients when compared with non-AS individuals

Table S1. Crude and multivariable Cox regression analyses for the relative risks of various IMIDs occurring after the index date in AS patients when compared with non-AS individuals

| **IMID** | Crude | | |  | Adjusted variables | | | | | | | | | | | | | | | | | | |
| --- | --- | --- | --- | --- | --- | --- | --- | --- | --- | --- | --- | --- | --- | --- | --- | --- | --- | --- | --- | --- | --- | --- | --- |
|  |  |  |  |  | Age | | |  | Age and sex | | |  | Age, sex and medications | | |  | Age, sex, medications and the frequency of visits | | |  | Age, sex, medications, the frequency of visits and CCI | | |
|  | HR (95% CI) | P | AIC |  | HR (95% CI) | P | AIC |  | HR (95% CI) | P | AIC |  | HR (95% CI) | P | AIC |  | HR (95% CI) | P | AIC |  | HR (95% CI) | P | AIC |
| **AAU** | 16.85 (14.87–19.08) | <0.01 | 23,617 |  | 16.86 (14.88–19.10) | <0.01 | 23,613 |  | 16.85 (14.88–19.09) | <0.01 | 23,604 |  | 12.85 (10.81–15.28) | <0.01 | 23,565 |  | 9.07 (7.52–10.94) | <0.01 | 22,857 |  | 9.03 (7.48–10.89) | <0.01 | 22,858 |
| **Psoriasis** | 3.75 (3.16–4.46) | <0.01 | 16,775 |  | 3.77 (3.18–4.48) | <0.01 | 16,743 |  | 3.77 (3.18–4.48) | <0.01 | 16,673 |  | 2.61 (1.98–3.43) | <0.01 | 16,509 |  | 1.70 (1.25–2.32) | <0.01 | 16,171 |  | 1.69 (1.24–2.29) | <0.01 | 16,167 |
| **IBD** | 8.19 (3.45–19.44) | <0.01 | 503 |  | 8.28 (3.49–19.66) | <0.01 | 500 |  | 8.28 (3.49–19.66) | <0.01 | 502 |  | 2.11 (0.39–11.48) | 0.39 | 201 |  | 1.75 (0.34–9.10) | 0.50 | 492 |  | 1.75 (0.34–9.13) | 0.50 | 494 |
| **CD** | 7.91 (1.77–35.35) | 0.01 | 172 |  | 8.00 (1.79–35.75) | 0.01 | 172 |  | 8.01 (1.79–35.79) | 0.01 | 172 |  | 8.81 (1.54–50.30) | 0.01 | 179 |  | 7.02 (1.23–39.97) | 0.03 | 175 |  | 6.88 (1.21–38.97) | 0.03 | 175 |
| **UC** | 9.57 (3.47–26.42) | <0.01 | 354 |  | 9.67 (3.50–26.70) | <0.01 | 355 |  | 9.66 (3.50–26.66) | <0.01 | 354 |  | 0.38 (0.05–2.81) | 0.34 | 348 |  | 0.35 (0.05–2.44) | 0.29 | 346 |  | 0.33 (0.05–2.37) | 0.27 | 342 |
| **SLE** | 7.94 (4.92–12.82) | <0.01 | 1,647 |  | 7.90 (4.89–12.75) | <0.01 | 1,643 |  | 8.06 (4.99–13.01) | <0.01 | 1,575 |  | 1.08 (0.57–2.04) | 0.82 | 1,208 |  | 0.91 (0.48–1.73) | 0.77 | 1,176 |  | 0.93 (0.49–1.79) | 0.84 | 1,172 |
| **SS** | 12.62 (9.34–17.06) | <0.01 | 3,998 |  | 12.83 (9.49–17.34) | <0.01 | 3,959 |  | 13.09 (9.68–17.70) | <0.01 | 3,840 |  | 2.33 (1.59–3.41) | <0.01 | 3,019 |  | 2.06 (1.40–3.03) | <0.01 | 2,921 |  | 2.05 (1.39–3.01) | <0.01 | 2,908 |
| **RA** | 20.11 (15.92–25.40) | <0.01 | 6,897 |  | 20.24 (16.02–25.57) | <0.01 | 6,889 |  | 20.67 (16.36–26.12) | <0.01 | 6,836 |  | 0.80 (0.57–1.13) | 0.21 | 5,410 |  | 0.69 (0.49–0.97) | 0.03 | 5,286 |  | 0.70 (0.50–0.99) | 0.04 | 5,286 |
| **SSc** | 9.51 (3.67–24.65) | <0.01 | 405 |  | 9.59 (3.70–24.87) | <0.01 | 405 |  | 9.59 (3.70–24.87) | <0.01 | 406 |  | 1.84 (0.51–6.65) | 0.35 | 353 |  | 1.65 (0.45–6.02) | 0.45 | 352 |  | 1.66 (0.46–6.03) | 0.44 | 354 |
| **DMtis** | 5.50 (1.66–18.28) | 0.01 | 289 |  | 5.57 (1.68–18.51) | 0.01 | 288 |  | 5.57 (1.68–18.52) | 0.01 | 289 |  | 1.24 (0.30–5.11) | 0.77 | 228 |  | 1.02 (0.24–4.27) | 0.98 | 218 |  | 0.95 (0.22–4.02) | 0.94 | 215 |
| **PM** | 10.32 (1.45–73.28) | 0.02 | 98 |  | 10.41 (1.47–73.93) | 0.02 | 98 |  | 10.41 (1.47–73.93) | 0.02 | 100 |  | 10.94 (1.34–89.68) | 0.03 | 99 |  | 8.30 (1.00–68.81) | 0.049 | 98 |  | 7.96 (0.97–65.56) | 0.053 | 98 |
| **TAO** | 16.62 (2.78–99.52) | <0.01 | 115 |  | 16.68 (2.79–99.92) | <0.01 | 117 |  | 16.69 (2.79–99.95) | <0.01 | 119 |  | 9.55 (0.87–105.14) | 0.07 | 123 |  | 6.63 (0.60–73.21) | 0.12 | 119 |  | 6.75 (0.61–75.12) | 0.12 | 121 |
| **BD** | 57.36 (19.68–167.14) | <0.01 | 534 |  | 57.00 (19.56–166.10) | <0.01 | 533 |  | 57.03 (19.57–166.18) | <0.01 | 535 |  | 24.24 (6.63–88.57) | <0.01 | 475 |  | 19.52 (5.30–71.91) | <0.01 | 467 |  | 20.37 (5.54–74.85) | <0.01 | 467 |
| **Pemphigus** | 5.62 (1.03–30.77) | 0.05 | 145 |  | 5.77 (1.05–31.60) | 0.04 | 141 |  | 5.76 (1.05–31.58) | 0.04 | 142 |  | 0.97 (0.07–13.48) | 0.98 | 119 |  | 0.95 (0.07–13.24) | 0.97 | 121 |  | 0.96 (0.07–13.85) | 0.97 | 122 |
| **Sarcoidosis** | 8.25 (4.30–15.81) | <0.01 | 884 |  | 8.24 (4.30–15.80) | <0.01 | 886 |  | 8.24 (4.30–15.80) | <0.01 | 888 |  | 7.40 (2.98–18.42) | <0.01 | 896 |  | 5.59 (2.24–13.99) | <0.01 | 876 |  | 5.39 (2.15–13.48) | <0.01 | 871 |
| **Vitiligo** | 2.36 (1.47–3.78) | <0.01 | 2,916 |  | 2.38 (1.49–3.82) | <0.01 | 2,898 |  | 2.38 (1.49–3.82) | <0.01 | 2,898 |  | 1.77 (0.82–3.80) | 0.14 | 2,881 |  | 1.50 (0.71–3.20) | 0.29 | 2,813 |  | 1.55 (0.73–3.32) | 0.26 | 2,812 |

Abbreviations: IMID, immune-mediated inflammatory disease; AS, ankylosing spondylitis; HR, hazard ratio; CI, confidence interval; P, p-value; AIC, Akaike information criterion; AAU, acute anterior uveitis; IBD, inflammatory bowel disease; CD, Crohn’s disease; UC, ulcerative colitis; SLE, systemic lupus erythematosus; SS, Sjögren's syndrome; RA, rheumatoid arthritis; SSc, systemic sclerosis; DMtis, dermatomyositis; PM, polymyositis; TAO, thromboangiitis obliterans; BD, Behcet’s disease.
