# Supplementary material for: Risk of immune-mediated inflammatory diseases in newly diagnosed ankylosing spondylitis patients: a population-based matched cohort study
Source: Arthritis Res Ther. 2019 Aug 29;21:196. doi: 10.1186/s13075-019-1980-1 (PMC6716905; doi:10.1186/s13075-019-1980-1)
Supplement: Supplementary file 2 — Table S2. Crude and multivariable Cox regression analyses for the relative risks of developing various IMIDs excluding those with IMID within three months after the index date in AS patients when compared with non-AS individuals. (DOCX 24 kb) [file 13075_2019_1980_MOESM2_ESM.docx]

**Risk of Immune-mediated Inflammatory Diseases in Newly-diagnosed Ankylosing Spondylitis Patients: A Population-based Matched Cohort Study**

Hsin-Hua Chen, Wen-Cheng Chao, Yi-Hsing Chen, Tsu-Yi Hsieh, Kuo-Lung Lai, Yi-Ming Chen, Wei-Ting Hung, Ching-Tsai Lin, Chih-Wei Tseng, Ching-Heng Lin

**Additional file 2.** Crude and multivariable Cox regression analyses for the relative risks of various IMIDs excluding those with IMID within three months after the index date in AS patients when compared with non-AS individuals

Table S2. Crude and multivariable Cox regression analyses for the relative risks of developing various IMIDs excluding those with IMID within three months after the index date in AS patients when compared with non-AS individuals

| IMID | Crude | | |  | Adjusted variables | | | | | | | | | | | | | | | | | | |
| --- | --- | --- | --- | --- | --- | --- | --- | --- | --- | --- | --- | --- | --- | --- | --- | --- | --- | --- | --- | --- | --- | --- | --- |
|  |  |  |  |  | Age | | |  | Age and sex | | |  | Age, sex and medications | | |  | Age, sex, medications and the frequency of visits | | |  | Age, sex, medications, the frequency of visits and CCI | | |
|  | HR (95% C.I.) | P | AIC |  | HR (95% C.I.) | P value | AIC |  | HR (95% C.I.) | P | AIC |  | HR (95% C.I.) | P | AIC |  | HR (95% C.I.) | P | AIC |  | HR (95% C.I.) | P | AIC |
| **AAU** | 19.31 (16.96–21.98) | <0.01 | 22,201 |  | 19.32 (16.97–22.00) | <0.01 | 22,198 |  | 19.31 (16.96–21.99) | <0.01 | 22,189 |  | 14.65 (12.28–17.49) | <0.01 | 22,154 |  | 10.98 (9.10–13.25) | <0.01 | 21,598 |  | 10.93 (9.05–13.20) | <0.01 | 21,600 |
| **Psoriasis** | 3.20 (2.63–3.90) | <0.01 | 13,971 |  | 3.22 (2.65–3.92) | <0.01 | 13,938 |  | 3.22 (2.65–3.92) | <0.01 | 13,883 |  | 1.69 (1.21–2.37) | <0.01 | 13,735 |  | 1.41 (1.01–1.96) | 0.045 | 13,634 |  | 1.41 (1.01–1.96) | 0.04 | 13,635 |
| **IBD** | 6.41 (2.52–16.29) | <0.01 | 458 |  | 6.51 (2.56–16.54) | <0.01 | 455 |  | 6.50 (2.56–16.53) | <0.01 | 455 |  | 0.36 (0.06–2.22) | 0.27 | 445 |  | 0.35 (0.06–2.06) | 0.24 | 440 |  | 0.34 (0.06–2.03) | 0.24 | 441 |
| **CD** | 2.68 (0.30–23.93) | 0.38 | 126 |  | 2.74 (0.31–24.52) | 0.37 | 123 |  | 2.74 (0.31–24.52) | 0.37 | 125 |  | 0.75 (0.03–20.26) | 0.86 | 131 |  | 0.65 (0.03–15.76) | 0.79 | 130 |  | 0.65 (0.03–15.82) | 0.79 | 131 |
| **UC** | 9.57 (3.47–26.42) | <0.01 | 354 |  | 9.67 (3.50–26.70) | <0.01 | 355 |  | 9.66 (3.50–26.66) | <0.01 | 354 |  | 0.38 (0.05–2.81) | 0.34 | 348 |  | 0.35 (0.05–2.44) | 0.29 | 346 |  | 0.33 (0.05–2.37) | 0.27 | 342 |
| **SLE** | 6.42 (3.79–10.85) | <0.01 | 1,438 |  | 6.38 (3.77–10.80) | <0.01 | 1,436 |  | 6.51 (3.85–11.02) | <0.01 | 1,377 |  | 0.84 (0.42–1.69) | 0.63 | 1,044 |  | 0.82 (0.41–1.65) | 0.58 | 1,045 |  | 0.81 (0.40–1.64) | 0.56 | 1,042 |
| **SS** | 8.55 (6.11–11.98) | <0.01 | 3,291 |  | 8.72 (6.23–12.22) | <0.01 | 3,250 |  | 8.88 (6.34–12.44) | <0.01 | 3,172 |  | 1.44 (0.94–2.20) | 0.09 | 2,408 |  | 1.31 (0.85–2.01) | 0.22 | 2,376 |  | 1.29 (0.84–1.99) | 0.25 | 2,369 |
| **RA** | 16.27 (12.73–20.79) | <0.01 | 6,036 |  | 16.41 (12.84–20.98) | <0.01 | 6,024 |  | 16.81 (13.15–21.48) | <0.01 | 5,966 |  | 0.52 (0.38–0.73) | 0.00 | 4,556 |  | 0.46 (0.33–0.65) | <0.01 | 4,509 |  | 0.47 (0.34–0.65) | <0.01 | 4,510 |
| **SSc** | 9.51 (3.67–24.65) | <0.01 | 405 |  | 9.59 (3.70–24.87) | <0.01 | 405 |  | 9.59 (3.70–24.87) | <0.01 | 406 |  | 1.84 (0.51–6.65) | 0.35 | 353 |  | 1.65 (0.45–6.02) | 0.45 | 352 |  | 1.66 (0.46–6.03) | 0.44 | 354 |
| **DMtis** | 2.80 (0.59–13.18) | 0.19 | 243 |  | 2.84 (0.60–13.40) | 0.19 | 242 |  | 2.84 (0.60–13.40) | 0.19 | 240 |  | 0.42 (0.07–2.57) | 0.35 | 184 |  | 0.36 (0.05–2.31) | 0.28 | 182 |  | 0.29 (0.04–1.99) | 0.21 | 176 |
| **PM** | 10.32 (1.45–73.28) | 0.02 | 98 |  | 10.41 (1.47–73.93) | 0.02 | 98 |  | 10.41 (1.47–73.93) | 0.02 | 100 |  | 10.94 (1.34–89.68) | 0.03 | 99 |  | 8.30 (1.00–68.81) | 0.049 | 98 |  | 7.96 (0.97–65.56) | 0.054 | 98 |
| **TAO** | 16.62 (2.78–99.52) | <0.01 | 115 |  | 16.68 (2.79–99.92) | <0.01 | 117 |  | 16.69 (2.79–99.95) | <0.01 | 119 |  | 9.55 (0.87–105.14) | 0.07 | 123 |  | 6.63 (0.60–73.21) | 0.12 | 119 |  | 6.75 (0.61–75.12) | 0.12 | 121 |
| **BD** | 69.95 (20.69–236.42) | <0.01 | 462 |  | 69.47 (20.55–234.80) | <0.01 | 462 |  | 69.56 (20.58–235.13) | <0.01 | 463 |  | 31.49 (7.60–130.44) | <0.01 | 410 |  | 26.20 (6.27–109.45) | <0.01 | 405 |  | 27.24 (6.54–113.53) | <0.01 | 406 |
| **Pemphigus** | 5.62 (1.03–30.77) | 0.05 | 145 |  | 5.77 (1.05–31.60) | 0.04 | 141 |  | 5.76 (1.05–31.58) | 0.04 | 142 |  | 0.97 (0.07–13.48) | 0.98 | 119 |  | 0.95 (0.07–13.24) | 0.97 | 121 |  | 0.96 (0.07–13.85) | 0.97 | 122 |
| **Sarcoidosis** | 7.10 (3.53–14.28) | <0.01 | 791 |  | 7.09 (3.52–14.26) | <0.01 | 793 |  | 7.09 (3.52–14.26) | <0.01 | 795 |  | 7.99 (3.17–20.14) | <0.01 | 802 |  | 6.36 (2.49–16.23) | <0.01 | 794 |  | 6.09 (2.38–15.58) | <0.01 | 786 |
| **Vitiligo** | 2.25 (1.35–3.73) | <0.01 | 2,612 |  | 2.28 (1.37–3.78) | <0.01 | 2,592 |  | 2.28 (1.37–3.78) | 0.00 | 2,593 |  | 1.64 (0.72–3.71) | 0.24 | 2,582 |  | 1.41 (0.63–3.20) | 0.41 | 2,576 |  | 1.42 (0.63–3.22) | 0.40 | 2,574 |

Abbreviations: IMID, immune-mediated inflammatory disease; AS, ankylosing spondylitis; HR, hazard ratio; CI, confidence interval; P, p-value; AIC, Akaike information criterion; AAU, acute anterior uveitis; IBD, inflammatory bowel disease; CD, Crohn’s disease; UC, ulcerative colitis; SLE, systemic lupus erythematosus; SS, Sjögren's syndrome; RA, rheumatoid arthritis; SSc, systemic sclerosis; DMtis, dermatomyositis; PM, polymyositis; TAO, thromboangiitis obliterans; BD, Behcet’s disease.
